# Development of a Composite Lifestyle Index and Its Relationship to Quality of Life Improvement: The CLI Pilot Study

**DOI:** 10.5402/2013/481030

**Published:** 2012-11-04

**Authors:** Thomas L. Lenz, Nicole D. Gillespie, Jessica J. Skradski, Laura K. Viereck, Kathleen A. Packard, Michael S. Monaghan

**Affiliations:** Department of Pharmacy Practice, Creighton University, 2500 California Plaza, Omaha, NE 68178, USA

## Abstract

An important component to optimal health is quality of life (QOL). Several healthy lifestyle behaviors have independently shown to improve QOL. The simultaneous implementation of multiple lifestyle behaviors is thought to be difficult, and the current literature lacks the assessment of multiple lifestyle behaviors simultaneously with respect to the effect on QOL. This current pilot study sought to develop a method to quantify multiple lifestyle behaviors into a single index value. This value was then measured with QOL for a possible correlation. The results showed that it is possible to convert multiple raw healthy lifestyle data points into a composite value and that an improvement in this value correlates to an improved QOL. After 12 months of participation in a cardiovascular risk reduction program, study participants (*N* = 35) demonstrated a 37.4% (*P* < 0.001) improvement in the composite lifestyle index (CLI). The improved CLI demonstrated a correlation with a statistically significant improvement in how participants rated their overall health in 12 months (*r* = 0.701, *P* < 0.001) as well as the number of self-reported unhealthy days per month in 12 months (*r* = −0.480, *P* = 0.004).

## 1. Introduction

Since 1948, the World Health Organization has defined health not only by the absence of disease or infirmity, but also as a state of complete physical, mental, and social well-being [1]. This definition implies that research outcomes should not only measure disease outcomes, but also quality of life outcomes. Measuring health-related quality of life provides a means of identifying and monitoring the impact of interventions on the physical and mental health of individuals as they themselves perceive this impact [2]. Quality of life may be measured objectively based on functioning or health status and subjectively based on one's own perception of health [3]. A number of lifestyle modifications including adequate nutrition, increased physical activity, adequate sleep, proper stress management, limited alcohol consumption, and tobacco cessation have been independently shown to have a positive effect on an individual's quality of life [2–13]. 

It is often assumed that initiating multiple behavior changes at the same time can become overwhelming for individuals and lead to decreased adherence. However, a recent study has shown that patients are able to effectively incorporate and maintain a number of lifestyle modifications initiated concomitantly [11]. The PREMIER clinical trial showed that participants could effectively incorporate and sustain multiple lifestyle changes to lower blood pressure risk and decrease cardiovascular risk. Lifestyle modifications successfully implemented included weight loss, reduced sodium intake, increased physical activity, limited alcohol consumption, and the dietary approaches to stop hypertension (DASH) diet [11]. Although no data currently exists, it may be advantageous for individuals with chronic medical conditions to be provided with a tool that can tract the implementation of multiple lifestyle behaviors simultaneously. This may provide the user with a more global view of the success of the implementation of overall healthy lifestyle behaviors rather than only focusing on one or two behaviors.

It may be important to educate patients about the direct health benefits from lifestyle-related activities as well as the quality of life benefits of a healthy lifestyle. It is currently unknown if an individual who participates in multiple lifestyle behaviors simultaneously will increase quality of life, even if the optimal level of each activity is not achieved. 

The objectives of this pilot study were to (1) develop a method that produces a single index value for multiple lifestyle behaviors that are implemented simultaneously (composite lifestyle index (CLI)), (2) measure the CLI versus a quality of life tool to see if a correlation relationship exists, and (3) use the results as a method to estimate a goal CLI that can be used in clinical practice. The study was approved by the Creighton University Institutional Review Board prior to initiation.

## 2. Materials and Methods

### 2.1. Study Participants

In 2008, a comprehensive cardiovascular risk reduction program (CVRRP) was developed at the private Midwestern University in the United States to curb the progression of cardiovascular disease in its employees [14]. The program offers the participants an individualized lifestyle modification program that targets several behaviors including physical activity, nutrition, weight control, sleep success, stress reduction, alcohol intake, and tobacco cessation. To be eligible for the program, the participant must be a university employee and have an existing diagnosis of hypertension, dyslipidemia, diabetes, or a combination thereof. Each participant must meet one-on-one with a clinical pharmacist no fewer than twelve times per year. During the meetings, a baseline risk assessment is completed, personalized lifestyle programs are developed, barriers to progress are addressed, and interventions are made as necessary. Several tools are used to improve awareness, education, adherence, and communication in the program. These tools include a lifestyle journal, nutrition diary, pedometer, home blood pressure monitor, exercise facility incentive, newsletter, blog site, and a support group. A detailed description of the program and examples of the tools used in the CVRRP have been published previously [14, 15]. The subjects described in this paper are a cohort of 35 participants (6 males/29 females) who participated in their first year of the program between September 2009 and September 2010. 

### 2.2. Composite Lifestyle Index (CLI)

 The CLI was developed by measuring six lifestyle components (i.e., physical activity, fruit and vegetable consumption, adequate sleep, stress management, alcohol consumption, and tobacco use) and converting the raw value to an index value. The quantifiable raw data of each of the 6 lifestyle components was converted to an index value on a scale of 0 to 10 or 1 to 10 (depending on the lifestyle activity), with 10 being optimal. To obtain a composite index, the index values earned from each of the 6 lifestyle components were added together for a possible composite index between 5 and 60 points. For each component, except for stress, the maximal index value assigned to the raw data was based on the optimal recommendation for that activity published in the respective practice guidelines. Raw data that was less than optimal was assigned index values that appeared to be reasonable for clinical application. The CLI calculation tables are shown in the appendix. 

#### 2.2.1. Physical Activity Index

The United States Department of Health and Human Services (USDHHS) recommends that adults participate in 150 minutes per week of moderate intensity physical activity, 75 minutes of vigorous activity per week, or 115 minutes of combined intensity physical activity per week [6]. Study participants who achieved this level of physical activity or greater received a score of 10 on the physical activity index. Percentages of this amount (at 10% increments) were then used to assign an index value to participants who achieved less than the recommended amount of physical activity. Participants who achieved 90–99% of the recommended amount of physical activity received an index value of 9, those who achieved 80–89% of this amount received an index value of 8, and so on.

#### 2.2.2. Fruit and Vegetable Consumption Index

The USDHHS recommends that adults consume an average of eight to ten combined servings of fruits and vegetables each day [12]. Study participants who consumed ten or more combined servings per day received a value of 10 on the fruit/vegetable index. Each serving less than 10 was subsequently recorded with the same index value. For example, 9 combined servings were indexed as a 9, 8 combined servings were indexed as an 8, and so on. It should be noted that prior to the study, the participants were provided a guide to help measure accurate serving sizes.

#### 2.2.3. Sleep Index

The CDC recommends that adults obtain an average of 7 to 9 hours of sleep each night [8]. Study participants who achieved this amount received a score of 10 on the sleep index. Participants who recorded more or less than the recommended amount subsequently received fewer points. The sleep index value decreased by 1 point for each 0.5 hour of less sleep or 1 hour of more sleep recorded. Less than 3 hours or more than 17 hours were recorded as 1 point.

#### 2.2.4. Stress Index

 No single data point to measure daily stress level currently exists that could be used for the purposes of the CVRRP or this study. Therefore, a scale to measure daily stress was created for participants in the CVRRP that could be used in their lifestyle journal and subsequently for the stress index. The scale asks the participants to reflect on their overall stress level at the end of the day and to rate it on the following scale:

 1 = low stress (feeling calm and in control)
 2
 3 = moderate stress
 4
 5 = high stress (feeling frantic and out of control).

Study participants who rated their stress level as a “1” on the stress scale were assigned a 10 on the stress index. Subsequently, the value on the stress index was decreased by 1 value for every 0.5 increase on the stress scale.

#### 2.2.5. Alcohol Consumption Index

The USDHHS recommends that adults consume no more than a moderate amount of alcohol each day. Moderate alcohol consumption is defined as up to two drinks per day for men and up to one drink per day for women [12]. Research has shown that frequent and high-quantity alcohol consumption is generally related to poorer health-related quality of life, and frequent. Low quantity consumption relates to a higher overall health-related quality of life [10]. High-quantity consumption was defined as 5 or more drinks per day [10]. Additionally, it is recommended that nondrinkers refrain from initiating alcohol consumption for the sole purpose of health benefits [12].

Quantifying alcohol consumption takes into account both the amount of alcohol consumed per episode and the frequency of episodes per week. Therefore, it is difficult to obtain a single index value for the alcohol index. To simplify the index value, the study participants who consumed moderate amounts of alcohol or less, as defined by the USDHHS, received 10 points on the alcohol index. Those who consumed more than moderate amounts received lower values, with 5 or more drinks/day for men and 4 or more drinks/day for women being the lowest values that could be earned. Because the number of drinks consumed per day can only be quantified as whole drinks in a practical manner, an alcohol index between 4 and 9 was not awarded.

#### 2.2.6. Tobacco Use Index

 The negative consequences of tobacco use have long been reported, and as a result, it is recommended that individuals abstain from smoking and tobacco use without exceptions [16]. Therefore, scoring the tobacco index was simplified by awarding individuals who do not use tobacco with a value of 10 and those who do use tobacco with a value of 0. This is the only lifestyle component in this pilot study to assign an individual lifestyle index value of 0. 

### 2.3. Quality of Life Measurement

The Centers for Disease Control and Prevention (CDC) health related quality of life (HRQOL) questionnaire is a statistically valid four-question survey that has been used by the CDC to measure population health-related quality of life since 1993 as a part of the Behavioral Risk Factor Surveillance System (BRFSS) [17]. The CDC uses a set of questions called the “Healthy Days Measures” to assess quality of life. These questions include the following.

- Question 1: “Would you say that in general your health is excellent, very good, good, fair or poor?”
- Question 2: “Now thinking about your physical health, which includes physical illness and injury, how many days during the past 30 days was your physical health not good?”
- Question 3: “Now thinking about your mental health, which includes stress, depression, and problems with emotions, how many days during the past 30 days was your mental health not good?”
- Question 4: “During the past 30 days, approximately how many days did poor physical or mental health keep you from doing your usual activities, such as self-care, work, or recreation?”

Unhealthy days are an estimate of the overall number of days during the previous 30 days when the respondent felt that either his or her physical or mental health was not good. To obtain this estimate, responses to questions 2 and 3 are combined to calculate a summary index of the overall unhealthy days, with a logical maximum of 30 unhealthy days [17].

### 2.4. Procedures

Participants in the study met with a clinical pharmacist member of the CVRRP team at the beginning of the program (baseline) and at least one time each month for the first six months of the program. Each participant completed a paper-based CDC HRQOL survey before beginning the program and then one time each month for the first six months. On a daily basis, each participant recorded his/her lifestyle activities in a lifestyle journal. For the purposes of this study, the information collected from the lifestyle journal included the number of minutes of physical activity per day, the number of servings of fruits and vegetables consumed per day, the number of hours of sleep per night, the number of alcoholic drinks consumed per day, the average perceived stress level per day, and if tobacco was used. This raw data was then averaged and converted to a composite lifestyle index (CLI) using the tables shown in the Appendix. The monthly CDC HRQOL values and the lifestyle indices were recorded for each lifestyle component as for well as the CLI. 

### 2.5. Statistical Analysis

To determine the correlation between the raw lifestyle component value and the CLI, the Spearman rank correlation coefficient (Spearman's rho) was used. The changes over time in the individual lifestyle index, CLI, and CDC HRQOL values were calculated using the Friedman test with the Bonferroni correction for multiple comparisons completed as appropriate with *P* values <0.017 were considered statistically significant. Additionally, the Spearman's rho was used to calculate the correlation between the CLI and the CDC HRQOL.

## 3. Results

The average age of the 35 participants at the beginning of the study was 50.7 years, and 10 participants had an existing diagnosis of diabetes, 22 had hypertension, and 20 had dyslipidemia. Study participants were followed for 1 year.

As stated in the above mentioned procedure, data was collected on a monthly basis for the first six months of the individual's participation in the CVRRP. After the study began, it was decided to also collect data at the 12-month time point and use it for comparison purposes to the baseline data. Therefore, participants were asked to complete the CDC HRQOL questionnaire at 12 months, and data from the participant's lifestyle journal was extracted for the month leading up to the 12-month visit to obtain the raw individual lifestyle component data.

Upon initial analysis of the data, it was discovered that of the 35 study participants, only 3 were currently consuming alcohol, and only 1 was a current tobacco user. As a result, there was not enough data on these two lifestyle components to include them in the analysis. Therefore, only the physical activity, fruit/vegetable, sleep, and stress components were included in the CLI and the analysis. This resulted in a final total CLI range of 4–40, rather than 5–60. 

The first step in the analysis was to measure the correlation between the lifestyle activity raw data and its conversion to the individual lifestyle index. The Spearman rank correlation coefficient showed that at each month the individual lifestyle index was significantly correlated (*P* < 0.001) with the raw data from each individual lifestyle component (physical activity, fruit/vegetable consumption, stress, and sleep).

Table 1 shows the change over time of the individual lifestyle index as well as the CLI. The CLI was shown to be significantly improved at both the 6-month (*P* < 0.001) and 12-month (*P* < 0.001) time periods compared with baseline. When looking at the individual lifestyle index, it appears that improvements in both physical activity and stress level contributed most towards the improvements in the CLI.

The changes over time with regards to the CDC HRQOL questionnaire showed a statistically significant improvement in how participants rated their overall health (Question 1) at baseline versus 6 months (3.21 versus 2.79, *P* = 0.003) and at baseline versus 12 months (3.21 versus 2.67, *P* = 0.005). Additionally, a summary index of the number of unhealthy days per month (question 2 + question 3) showed a statistically significant improvement from baseline at 12 months (−5.5 days/month, *P* = 0.007) but not at 6 months compared with baseline (−1.7 days/month, *P* = 0.113). 

After one year in the program, a statistically significant positive correlation between the CLI and how participants rated their general health was observed (*r* = 0.701, *P* < 0.001). Also during this time period, a statistically significant negative correlation between the CLI and the number of overall unhealthy days per month was observed (*r* = −0.480, *P* = 0.004). This demonstrates that a higher CLI correlates with better perceived health and with less unhealthy days each month. 

## 4. Discussion

The CLI pilot data presented in this paper demonstrates that it is possible to develop a scoring method that provides a single index value for multiple lifestyle behaviors that are implemented simultaneously and that this value can be correlated with improved quality of life. Studies have shown that lifestyle medicine components such as proper nutrition, exercise, adequate sleep, stress management, moderate alcohol consumption, and tobacco cessation measured individually can have a positive effect on quality of life improvement [2, 5, 7, 9, 10, 13]. However, research on the impact of quality of life from the simultaneous implementation of several lifestyle medicine components is currently lacking. Likewise, developing a single index value, such as the CLI, may be helpful for patients to holistically measure their healthy lifestyle activities.

For many people diagnosed with chronic conditions, feeling good, both physically and mentally, is a priority. Many times health care providers try to get their patients to implement healthy lifestyle activities to not only improve biometric health status, but to feel better as well. One of the difficulties that both providers and patients have is successful and sustained implementation of healthy lifestyle activities. Providers often tell patients to achieve the recommended amounts of each activity according to the practice guidelines. Because many patients fail to achieve the recommended amounts of each lifestyle activity simultaneously, they may feel as though they have failed to be successful with lifestyle activities, in general. What is currently unknown in the literature is the notion that if an individual implements multiple lifestyle behaviors simultaneously, but is not achieving the optimum level of each, can he/she still improve quality of life? The hypothesis when developing the CLI was that patients with chronic conditions do not need to achieve all healthy lifestyle behaviors at the recommended levels in order to improve overall quality of life. The authors feel as though the pilot study was successful in demonstrating that an improvement in the CLI can lead to an improvement in quality of life. Therefore, health care providers may be able to use the CLI with their patients as a method to track the simultaneous implementation of multiple healthy lifestyle behaviors to show global improvements and as a means to relate the concept of enhanced quality of life.

Several limitations were involved with this study. The number of participants was low with a relatively short assessment time period which may have made the statistical analysis underpowered. Also, participants in the CVRRP are asked on a daily basis to track several healthy lifestyle-related activities via a lifestyle journal. This self-reported tracking process is to be completed at the end of each day. The raw individual lifestyle component data for this study was based on the data recorded in each participant's lifestyle journal. Self-reporting can have limitations based on participant's truthfulness, recall, accuracy, and missing data. Of the 35 individuals enrolled in the study, complete matching data for CLI at baseline, 6 months, and 12 months was available in only 19 individuals.

Limitations existed in the individual lifestyle indices as well. The stress index was based on a scale developed for the CVRRP and not on a validated stress measurement tool. The fruit/vegetable index was intended to represent healthy eating but is only one component of healthy eating. Additionally, the fruit/vegetable index did not contain a possible score of “0” to represent an absence of daily fruit and vegetable intake. Finally, the sleep index only quantified the amount of sleep and not the quality of sleep.

Lastly, each individual lifestyle index was weighted the same as in the CLI calculation which may have affected the results. Previous research has shown that if just one of the six lifestyle components is achieved at the recommended level, quality of life is improved. Ongoing research of the CLI will address a weighted value for each individual lifestyle indices.

The third objective of the CLI pilot study was to use the results as a method to estimate a goal CLI that can be used in clinical research. Although the results showed that participants who achieved a CLI of at least 27.5 (37.4% improvement) were able to improve their quality of life, the information obtained from the pilot data was not sufficient enough to be able to definitively provide a specific CLI that patients should strive for that will lead to an improvement in quality of life. It was only able to show that improvements in the CLI of at least 37.4% in 12 months lead to an improved quality of life in the study participants. 

The authors, however, have developed patient goals based on the CLI that provide a starting point for practical use of the CLI. Including scoring for both alcohol consumption and tobacco use (excluded from the pilot study data), the CLI has a maximum value of 60. Patients in the CVRRP are recommended to set an initial CLI goal of 20 with a lifelong goal of 40 or more. Establishing these cut points seemed reasonable because a CLI of 20 is a realistic initial goal for most participants in the program, and a CLI of 40 required most participants to make several healthy behavior changes. The point of emphasis that is made to the CVRRP participants when using the CLI is that continual global improvements are important as is the sustainability of the CLI once it reaches at least 40. 

Data collection for the CLI is ongoing as more individuals are enrolled in the CVRRP. It is intended that future data analyses will include alcohol consumption and tobacco use. One change made to the CLI since the completion of the pilot study was the addition of a “0” to the fruit/vegetable index to represent an absence of any fruit or vegetable consumption. Additional analyses will attempt to measure if the CLI of suboptimal individual indices on multiple lifestyle activities shows similar increases in quality of life versus the CLI of an optimal level in just one activity. Additional analyses will also include quality of life assessments of the CLI when optimal levels in multiple activities are achieved versus the CLI obtained when an optimal level in only one activity is achieved.

## 5. Conclusions

 The CLI pilot study was able to show that raw individual lifestyle component data could be successfully converted to an individual lifestyle index and subsequently to a Composite Lifestyle Index (CLI). The CLI could then be correlated with an individual's quality of life measurement with a practical method for use in clinical practice. The pilot data showed that an increase in the CLI correlated with an improvement in the quality of life for individuals with a chronic medical condition.

## Figures and Tables

**Table 1:** Change over time in mean individual lifestyle indices and in CLI.

Component (*n*)	Baseline	6 Months	12 Months	Relative change over time: % (*P* value at 6 months versus baseline) % (*P* value at 12 months versus baseline)
Physical activity (*n* = 21)	3.67	7.76	7.24	111.4% (0.002) 97.3% (<0.001)
Fruit/vegetable (*n* = 20)	3.30	3.80	4.35	15.2% (0.115) 31.8% (0.025)
Sleep (*n* = 21)	9.10	9.10	9.48	0% (0.262) 4.2% (0.147)
Stress (*n* = 20)	4.50	7.50	6.85	66.7% (<0.001) 52.2% (0.001)
CLI (*n* = 19)	20.00	28.53	27.47	42.7% (<0.001) 37.4% (<0.001)

CLI: composite lifestyle index.

*P* < 0.017 is considered statistically significant.

**Table 2:** 

Value	Moderate activities (minutes/week)	Vigorous activities (minutes/week)	Combination activities (minutes/week)
10	150+	75+	115+
9	135–149	68–74	104–114
8	120–134	60–67	92–103
7	105–119	53–59	81–91
6	90–104	45–52	69–80
5	75–89	38–44	58–68
4	60–74	30–37	46–57
3	45–59	23–29	35–45
2	30–44	15–22	23–34
1	0–29	0–14	0–22

Physical activity index.

**Table 3:** 

Value	Combined fruit and vegetable servings (servings/day)
10	10
9	9
8	8
7	7
6	6
5	5
4	4
3	3
2	2
1	1

Fruit/vegetable index.

**Table 4:** 

Value	Sleep (hours/night)
10	7 to 9
9	6.5 to 7 or 9 to 10
8	6 to 6.5 or 10 to 11
7	5.5 to 6 or 11 to 12
6	5 to 5.5 or 12 to 13
5	4.5 to 5 or 13 to 14
4	4 to 4.5 or 14 to 15
3	3.5 to 4 or 15 to 16
2	3 to 3.5 or 16 to 17
1	Less than 3 or More than 17

Sleep index.

**Table 5:** 

Value	Stress scale (average/day)
10	1
9	1 to 1.4
8	1.5 to 1.9
7	2 to 2.4
6	2.5 to 2.9
5	3 to 3.4
4	3.5 to 3.9
3	4 to 4.4
2	4.5 to 4.9
1	5

Stress index.

**Table 6:** 

Value	Men (drinks/episode day)	Women (drinks/episode day)
10	0 to 2	0 to 1
9	NA	NA
8	NA	NA
7	NA	NA
6	NA	NA
5	NA	NA
4	NA	NA
3	3	2
2	4	3
1	5 or more	4 or more

Alcohol index.

**Table 7:** 

Value	Tobacco use
10	Is not using tobacco
0	Currently using tobacco

Tobacco index.

**Table 8:** 

Lifestyle activity	Index
Physical activity	
Nutrition	
Sleep	
Stress	
Alcohol consumption	
Tobacco use	

CLI	

## Appendix

### Composite lifestyle Index (CLI) Calculation

*Physical Activity Index.* On Table 2, find the average number of weekly minutes of physical activity during the previous 4 weeks and record the index value associated with that amount.

*Fruit/Vegetable Consumption Index.* On Table 3, find the average number of daily servings of combined fruits and vegetables consumed during the previous 4 weeks and record the index value associated with that amount.

*Sleep Index.* On Table 4, find the average number of hours of sleep per night during the previous 4 weeks and record the point value associated with that amount.

*Stress Index.* Using the stress scale in *Lifestyle Journal*, record the associated point value in Table 5 of the average daily stress scale score over the previous 4 weeks.

*Alcohol Consumption Index.* On Table 6, find the average number of alcoholic drinks consumed per day when alcohol consumption has occurred during the previous 4 weeks and record the point value associated with that amount. This average is the number of drinks consumed per episode rather than the average number of drinks consumed for each day of the previous month (such as counting days when alcohol consumption did not take place).

*Tobacco Use Index.* On Table 7, record a “10” if tobacco is not currently being used and a “0” if tobacco (ex. smoking or smokeless tobacco) is currently being used.

*Composite Lifestyle Index (CLI).* Record the Index value for each lifestyle activity in Table 8. Add the index values together to obtain the composite lifestyle index (CLI).
